# A cross-sectional study of the income sources of primary care health workers in the Democratic Republic of Congo

**DOI:** 10.1186/s12960-017-0185-4

**Published:** 2017-02-20

**Authors:** Rishma Maini, David R. Hotchkiss, Josephine Borghi

**Affiliations:** 1 London School of Hygiene and Tropical Medicine, 15-17 Tavistock Place, London, WC1H 9SH United Kingdom; 2 School of Public Health and Tropical Medicine, Tulane University, 1440 Canal Street, New Orleans, LA 70112 United States of America

**Keywords:** Remuneration, Income, DRC, Primary care, Health workers

## Abstract

**Background:**

In the Democratic Republic of Congo (DRC), the state system to remunerate health workers is poorly functional, encouraging diversification of income sources and corruption. Given the central role that health workers play in health systems, policy-makers need to ensure health workers are remunerated in a way which best incentivises them to provide effective and good quality services. This study describes the different sources and quantities of income paid to primary care health workers in Equateur, Maniema, Kasai Occidental, Province Orientale and Kasai Oriental provinces. It also explores characteristics associated with the receipt of different sources of income.

**Methods:**

Quantitative data on the income received by health workers were collected through baseline surveys. Descriptive statistics explored the demographic characteristics of health workers surveyed, and types and amounts of incomes received. A series of regression models were estimated to examine the health worker and facility-level determinants of receiving each income source and of levels received. Qualitative data collection was carried out in Kasai Occidental province to explore perceptions of each income source and reasons for receiving each.

**Results:**

Nurses made up the majority of workers in primary care. Only 31% received a government salary, while 75% reported compensation from user fees. Almost half of all nurses engaged in supplemental non-clinical activities. Receipt of government payments was associated with income from private practice and non-clinical activities. Male nurses were more likely to receive per diems, performance payments, and higher total remuneration compared to females. Contextual factors such as provincial location, presence of externally financed health programmes and local user fee policy also influenced the extent to which nurses received many income sources.

**Conclusions:**

The receipt of government payments was unreliable and had implications for receipt of other income sources. A mixture of individual, facility and geographical factors were associated with the receipt of various income sources. Greater co-ordination is needed between partners involved in health worker remuneration to design more effective financial incentive packages, reduce the fragmentation of incomes and improve transparency in the payment of workers in the DRC.

## Background

Health workers play a central role in the delivery of health care, and their remuneration influences their motivation and workplace performance [1–4]. Post conflict states present an interesting context for examining health worker remuneration as the state system of salary payment is often poorly functional, encouraging a diversification of income sources [5]. Donors may exacerbate income fragmentation by providing programme-related performance payments and per diems [6]. Evidence from these settings has shown that income received from different sources varies according to individual worker and health facility characteristics, with female workers receiving significantly less salary and total income than male workers of the same cadre in Sierra Leone [7], and rural workers having less access to user fee revenue and income from non-clinical activities to those in urban areas in Zimbabwe [8]. A study by Bertone et al. in four provinces in the Democratic Republic of Congo (DRC) identified individual, facility and provincial determinants of variation in total income received [9]. However, the Sierra Leone and Zimbabwe studies were descriptive and did not comprehensively examine the determinants of receiving each income source and none of these studies looked at how receipt of one income source affects the likelihood of receiving other sources, or compiled qualitative data to obtain more in-depth insights into remuneration practices. Moreover, Bertone and Witter have advocated for more empirical research on the overall revenue or “complex remuneration” of health workers, in order to devise effective incentive packages [10].

This study aims to address this gap by examining the remuneration structure of health workers in five provinces of the DRC,^1^ assessing the determinants of receiving income by source and the inter-dependency of different sources of income, as well as the determinants of total income received. Qualitative methods are also used to substantiate the quantitative findings and help to discover the processes and mechanisms that underpin the quantitative results.

### DRC context

In 2013, public investment in health was only 4.5% of the national budget falling far short of the Abuja commitment of 15% [11]. Although all public sector health workers should receive a salary and occupational risk allowance (or “prime de risque”) from the government, not everyone receives these. Services therefore rely heavily on cost recovery through user fees, with no accepted standard national tariff for consultations. Health workers have also become dependent on performance-based payments and/or per diems from external partners.

The public care system accounts for about half of all facilities in the country [12]. The basic unit of the primary care health system is the health zone [13]. Health zones are divided into health areas serving 10,000 to 15,000 people. Each health area should have a health centre providing an essential package of primary healthcare activities. Health centres equipped to carry out certain minor surgical operations are termed reference health centres. In the absence of a health centre or reference health centre, there is a health post.

## Methods

Health facility and health worker surveys were conducted as part of a baseline survey linked to an evaluation of a health systems strengthening programme funded by the Department for International Development (DfID) called ASSP (Accès Aux Soins de Santé Primaires) in April–May 2014 [Keating J, Hotchkiss D, Eisele T, Kitoto AT, Bertrand J. Evaluation of the impact of the ASSP project in the Democratic Republic of Congo, unpublished].

Data collection was carried out by data collectors hired from each of the provinces to ensure familiarity with the cultural context. Participation of health workers in the survey was voluntary. To minimise the potential for social desirability bias, the interviewer explained the purpose, confidentiality and anonymity of the study to each provider before seeking consent to begin the survey.

Surveys were carried out in Equateur, Kasai Occidental, Kasai Oriental, Province Orientale, and Maniema provinces in 105 intervention villages selected using probability proportional to size (PPS), and an equal number of control villages matched on geographic location and population size. In total, 210 facilities were selected and all workers providing clinical services and on duty the day of the survey were interviewed.

The health worker survey measured age, sex, cadre, marital status, educational attainment, number of years in their current position, and number of financial dependents. The survey identified income sources received and income levels adapted from the Health Worker Incentive Survey [14] for government payments (salaries, occupational risk allowances), performance payments and per diems from non-governmental partners, private clinical work, user fees from patients, informal payments^2^ or “gifts”, allowances, and income from non-clinical activities. Respondents were asked whether government payments were received on time, if there were delays receiving these payments, and amounts received compared to expectations. Income levels were recorded in Congolese Francs (FC). Recall was for “last month received” for all incomes with the exception of per diems which was for the “last year”. A facility survey was also carried out to measure the total number of staff, distance of the facility from the village, and the number of primary healthcare services provided. Both surveys were piloted in two health facilities in Kinshasa and one facility in Bas Congo.

Qualitative research was carried out in November 2014 in four urban and four rural health zones supported by ASSP in the province of Kasai Occidental that were not included in the survey. Two nurses (one female and one male) were purposively selected from a health centre in each health zone, making a total of 16 nurses. Interviews examined the sources and amounts of income received, and factors influencing their receipt and were audio recorded in French by RM and a local researcher. Hand written notes were also taken.

### Data analysis

Survey data were double entered into CSPro and imported into STATA 13.0 for analysis. Income data were converted into United States dollars (USD) using the exchange rate of 923 FC to 1 USD.^3^ Grubb’s test was applied to detect outliers in the income data, which were removed prior to analysis. Descriptive statistics were generated for health worker characteristics, receipt of income by source and mean and median income levels. The frequency of receipt of government payments and income compared to expectations are also reported. Logistic regressions examined facility and health worker characteristics associated with receiving a given income source and linear regressions identified determinants of the level of income received, measured as the log of income. Presence of the ASSP programme was included as an explanatory variable in all of the models. Health worker explanatory variables were health worker age, marital status, sex, cadre, education, years worked in position, and the number of financial dependents; facility-level factors were provincial location, urban-rural status, facility type, number of staff, distance from the nearest village, and the number of services offered. We also examined whether receipt of certain income sources affected the receipt of others. The hypothesised relationships between the independent variables and income sources are given in Appendix 1.

In total, 18 models were run and regression diagnostics applied and adjustments made to produce unbiased coefficients (Appendix 2). A general to specific regression specification method was used, excluding explanatory variables in a stepwise manner. All regressions were performed excluding any missing values (list-wise deletion) with clustering at the facility level.

Audio recordings of the interviews were transcribed and a coding system was developed by RM from the initial research themes and concepts that emerged during data collection. Data was managed using NVivo 10 and content analysis was used to identify key themes.

## Results

### Sample characteristics

Three facilities were private clinics and so did not meet the inclusion criteria of being public sector primary care facilities. This left 207 facilities for analysis.

Twenty three health workers did not meet the inclusion criteria, leaving 453 respondents for analysis. No health workers declined to participate in the survey.

Health workers were mainly located in rural facilities (80.6%) and were based in health centres (81.7%) (Table 1). Most workers were in Kasai Occidental followed by Maniema and Equateur. Most of the workers were in facilities located within 5 km of the nearest village (79.9%), and over 75% offered between six and nine services.

**Table 1:** Facility characteristics of sampled respondents

Facility characteristics of sampled (*n*) workers	Proportion of workers
	%
Facility location (*n =* 453)	
Rural	80.6
Urban	19.4
Province (*n =* 453)	
Equateur	23.0
Kasai Occidental	29.8
Kasai Oriental	5.7
Maniema	27.6
Province Orientale	13.9
Type of facility (*n =* 453)	
Health centre	81.7
Reference health centre	17.2
Health post	1.1
Distance of facility from the village (*n =* 443^a^)	
Less than 1 km	31.6
Between 1 and 5 km	48.3
Between 5 and 10 km	12.0
Greater than 10 km	8.1
Number of services provided by facility (*n =* 435^a^)	
3 to 5 services	12.2
6 to 9 services	76.1
Over 10 services	3.0
Total clinical staff present on the day (*n =* 453)	
1	13.3
2	34.0
3	23.8
4	16.8
5	6.6
6	4.0
7	1.6
Population catchment for area (*n =* 430^a^)	
Less than 5000	48.9
5000 to 10,000	21.6
10,001 to 15,000	17.4
Greater than 15,000	12.1

^a^Less than 453 due to missing values for those variables

Most respondents were male and between 30 and 45 years old (Table 2). Ninety percent of staff were nurses, and only four doctors were identified across all facilities. The majority had some secondary level education and a third had been to university. Most workers were married and had worked a median of 6 years in their current position.

**Table 2:** Characteristics of health workers

Characteristics	Proportion of all workers interviewed	Proportion of nurses
	%	%
Sex	(*n =* 453)	(*n =* 407)
Male	69.3	70.3
Female	30.7	29.7
Age	(*n =* 453)	(*n =* 407)
<30 years	11.5	12.3
30–44 years	59.7	60.7
45–60 years	26.1	24.6
>60 years	3.1	2.5
Marital status	(*n =* 447^a^)	(*n =* 407)
Married	90.4	91.8
Single	3.8	3.5
Widowed	3.4	2.5
Separated/divorced	2.2	2.0
Other	0.2	0.3
Education	(*n =* 453)	(*n =* 407)
Primary school	0.4	0.3
Secondary school	60.3	62.9
University/post-secondary school	33.1	35.1
Not specified	6.2	1.7
Position	(*n =* 453)	N/A
Doctor	0.9	
Nurse	89.8	
Laboratory worker	1.1	
Pharmacy worker	1.3	
Traditional birth attendant	2.9	
Auxiliaries, medical and nursing assistants (other non-qualified personnel)	4.0	
	*N*, mean, SE (median, IQR)	*N*, mean, SE (median, IQR)
Number of financial dependents	437^a^, 9, 4.56. (8, 6–12)	393^a^, 9, 4.63. (8, 6–12)
Years worked in current position	446^a^, 9, 8.72. (6, 3–12)	403^a^, 9, 8.68. (6, 3–11)

^a^Less than 453 for all workers or less than 407 for nurses due to missing values for those variables

The analysis of income sources and levels focuses on nurses.

### Income sources and levels

**Fig. 1:**
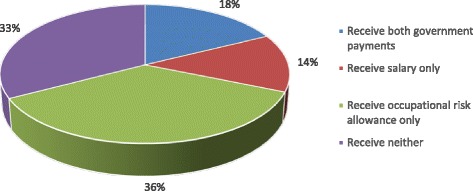
Proportion of nurses receiving: both government payments, one government payment only, or no government payments

Only one third of nurses reported receiving a salary while over half received the occupational risk allowance (Table 3, Fig. 1). A third of nurses did not receive any form of government payment and 18% received both an occupational risk allowance and a salary (Fig. 1). Of non-governmental payments, the most frequently reported were user fees, followed by per diems. Just under half of the sample (47%) reported receiving income from supplementary non-clinical activities. Of these most worked in agriculture (68%), followed by trade (28%). A minority of nurses (7%) reported receiving allowances for uniforms, housing or transport. Less than 10% of workers (*n* = 29) reported receiving income from private clinical practice, mostly practising at home (*n* = 24).

**Table 3:** Proportion of nurses receiving sources of income and mean and median values of income received

Source of income	Overall proportion of workers who received source of income	Median income per month among those receiving income in USD (IQR)	Mean income per month among those receiving income in USD (standard error)
Payments from government			
Salary from government (*n =* 407)	31.2%	52.76 (23–75)	58.06 (60.45)
Occupational risk allowance from government (*n =* 407)	53.8%	12.46 (11–16)	36.57 (73.38)
Payments from other sources			
Performance pay (*n =* 407)	24.1%	16.25 (9–46)	35.79 (48.81)
User fees (*n =* 406)	74.6%	19.50 (11–38)	71.02 (157.95)
Gifts/informal payments from patients (*n =* 406)	16.8%	4.60 (2–11)	8.73 (10.43)
Per diems (*n =* 406)	51.7%	4.06 (2–8)	8.56 (26.35)
Income from private clinical practice (*n =* 407)	7.1%	21.67 (11–54)^a^	34.02 (34.05)^a^
Income from supplemental (non-clinical) activities (*n =* 400)	46.8%	65.01 (33–114)^a^	119.27 (154.62)^a^
Total income (*n =* 300^a^)	N/A	85.05 (36–176)^a^	165.26 (227.55)^a^

N.B. For the occupational risk allowance, one outlier income was dropped from the analysis; no outliers were detected for any other income amount

^a^Greater than 10% of data missing as respondents had missing values for some of the amounts of income

The highest median monthly income was for non-clinical work outside the facility ($119), followed by the government salary ($58). The lowest median monthly income came from per diems ($9) and informal payments ($9). The median monthly income across all sources was $85 but the mean was almost double at $165.

**Fig. 2:**
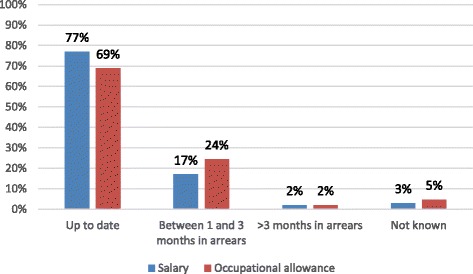
Frequency of government payments to nurses

Over two thirds of nurses receiving the salary and the occupational risk allowance payments reported they were paid on time. Seventeen percent of nurses reported receiving salaries between 1 and 3 months in arrears and 24% reported doing so for occupational risk allowances. Only 2% reported receiving salaries and/or occupational risk allowances more than 3 months in arrears (Fig. 2).

**Fig. 3:**
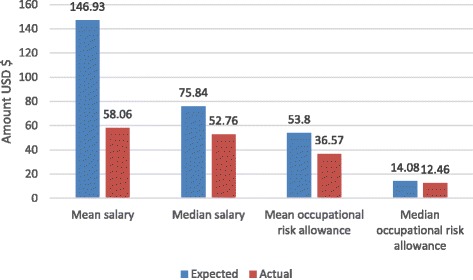
Median and mean amounts of expected and actual salary and occupational risk allowance for nurses

Despite the overall timeliness of payments, the amounts received from government were less than expected (Fig. 3).

Nurses complained more about the frequency of salary payments, than of occupational risk allowances, stating irregular salary payment with no set day of the month. Many had to regularly request an advance from the facility as they usually ran out of money before their next pay day. It was also common for nurses to borrow from their family or friends to pay the rent or school fees. Nurses reported huge variability in user fee revenue as it was dependent on the number of patients seen at the facility.

None of the in-depth interview respondents reported engaging in private practice or receiving performance payments from partners. A few reported receiving per diems for training or vaccination campaigns. Informal payments or gifts from patients were often in the form of soap, fabric, or food. All nurses were dissatisfied with total compensation received.

### Income determinants

The likelihood of receiving a salary increased with every year worked at the facility (OR 1.06, *p* < 0.000) and was greater for staff working in urban facilities (OR 2.48, *p* = 0.021). Nurses were more likely to receive a salary if they were in Equateur than Maniema (0.22, *p* = 0.014).

The odds of receiving the occupational allowance were greater if the nurse had more years of experience (OR 1.20, *p* < 0.000) and a higher number of dependents (OR 1.12, *p* = 0.001. The odds of receiving the occupational allowance were highest in Province Orientale (OR 9.58, *p* = 0.001) compared to Equateur, but lower in Kasai Occidental (OR 0.17, *p* < 0.000) or Kasai Oriental (OR 0.05, *p* < 0.000) (Table 4).

**Table 4:** Logistic regressions for salary and occupational risk allowance

	Odds ratio for dependent variables (SE)			
Explanatory variables	Salary		Occupational risk allowance	
	Full model	Reduced model	Full model	Reduced model
Years in position	1.06 (0.02)***	1.06 (0.02)***	1.19 (0.04)***	1.20 (0.04)*** (*p <* 0.001)
Kasai Occidental (vs Equateur)	1.48 (0.69)	1.46 (0.64)	0.17 (0.07)***	0.17 (0.07)*** (*p <* 0.001)
Kasai Orientale (vs Equateur)	1.02 (0.89)	0.71 (0.48)	0.03 (0.02)***	0.05 (0.04)*** (*p <* 0.001)
Maniema (vs Equateur)	0.20 (0.13)**	0.22 (0.14)*** (*p =* 0.014)	1.56 (0.76)	1.30 (0.63)
Province Orientale (vs Equateur)	0.69 (0.43)	1.05 (0.52)	11.07 (7.28)***	9.58 (6.33)*** (*p =* 0.001)
Population served	1.00 (0.00)**	1.00 (0.00)** (*p =* 0.043)	1.00 (0.00)	
Total personnel	1.43 (0.25)**		0.94 (0.19)	
Urban (vs rural)	1.90 (0.86)	2.48 (0.97)** (*p =* 0.021)	2.42 (1.33)	2.10 (0.91)*
Number of services	1.08 (0.12)		0.84 (0.09)	
Distance of facility from village	1.00 (0.03)		1.07 (0.04)*	
Reference heath centre (vs heath centre)	0.74 (0.36)		0.49 (0.24)	0.39 (0.20)*
Age	1.02 (0.02)		1.03 (0.02)	
Male (vs female)	0.92 (0.31)		0.66 (0.24)	
Number of dependents	0.97 (0.03)		1.11 (0.04)**	1.12 (0.04)*** (*p =* 0.001)
Married (vs not married)	1.10 (0.14)		0.67 (0.12)**	0.75 (0.12)*
University (vs school education)	0.77 (0.27)		1.07 (0.41)	
ASSP programme	0.70 (0.28)		0.58 (0.23)	
Received occupational allowance (salary model only)	1.03 (0.35)		–	–
Received salary (occupational risk allowance model only)	–	–	1.06 (0.39)	
Constant	0.04 (0.05)**	0.09 (0.04)***	0.37 (0.41)	0.21 (0.08)***
Pseudo *R* ^2^	0.17	0.14	0.35	0.33
Model *χ* ^2^	50.90***	46.98***	91.22***	77.56***
Number observations (*n*)	337	383	318	318

*p ≤ 0.1; **p ≤ 0.05; ***p ≤ 0.01

In the in-depth interviews, nurses who did not get a salary stated it was because they started working after the last census of workers in 2006, which was used as a basis for payroll. Some nurses felt health zone officials discriminated against workers from certain tribal or ethnic backgrounds in the payment of the occupational risk allowance.

According to the quantitative analysis, compared to Equateur, nurses in Maniema were more likely to receive all other sources of income, with the exception of per diems (Table 5). Only those with a higher number of dependents were significantly more likely to receive user fees (OR 1.07 *p* = 0.038).

**Table 5:** Logistic regressions for determinants of non-governmental sources of income

	User fees		Informal payments		Private payment		Non-clinical activities		Performance payments		Per diems	
Explanatory variables	Odds ratio (SE)											
	Full	Reduced	Full	Reduced	Full	Reduced	Full	Reduced	Full	Reduced	Full	Reduced
Years in position	1.02 (0.02)		1.03 (0.03)		1.02 (0.05)		0.97 (0.02)		1.02 (0.04)		0.99 (0.02)	
Kasai Occidental (vs Equateur)	0.52 (0.23)	0.87 (0.32)	3.13 (2.13)*	2.34 (1.17)*	8.72 (10.09)*	8.42 (8.88)**	0.69 (0.25)	0.65 (0.23)	2.62 (3.02)	2.69 (3.13)	1.29 (0.48)	1.24 (0.41)
Kasai Orientale (vs Equateur)	3.22 (3.86)	3.81 (2.94)*	5.84 (4.64)**	3.77 (2.37)**	1	1	0.99 (0.91)	0.46 (0.37)	1	1	3.67 (3.50)	2.02 (1.35)
Maniema (vs Equateur)	2.65 (1.58)	3.76 (1.73)***	23.82 (18.79)***	12.49 (7.39)***	21.87 (25.97)***	14.53 (16.24)**	3.59 (1.68)***	4.02 (1.81)***	158.86 (191.31)***	132.02 (147.97)***	0.50 (0.26)	0.56 (0.25)
Province Orientale (vs Equateur)	1.54 (0.89)	1.08 (0.47)	9.18 (5.93)***	5.76 (3.00)***	1	1	3.99 (2.27)**	4.42 (2.17)***	1	1	0.41 (0.20)*	0.37 (0.15)**
Population served	1.00 (0.00)		1.00 (0.00)		1.00 (0.00)		1.00 (0.00)		1.00 (0.00)**	1.00 (0.00)**	1.00 (0.00)	
Total personnel	1.12 (0.20)		0.69 (0.11)**	0.67 (0.10)**	0.83 (0.16)		0.79 (0.11)*	0.83 (0.12)	0.98 (0.21)		0.68 (0.11)**	0.72 (0.10)**
Urban (vs rural)	0.63 (0.32)		0.95 (0.48)		2.81 (1.85)	2.44 (0.99)**	0.51 (0.18)*	0.51 (0.15)**	1.17 (0.82)		1.37 (0.55)	
Number of services	0.98 (0.14)		0.96 (0.14)		0.90 (0.10)		1.35 (0.12)***	1.24 (0.11)**	1.71 (0.26)***	1.51 (0.22)***	1.24 (0.11)**	1.24 (0.09)**
Distance of facility from village	0.98 (0.03)		0.97 (0.05)		0.78 (0.07)**	0.88 (0.06)**	0.99 (0.03)		0.78 (0.05)***	0.79 (0.06)***	0.99 (0.03)	
Reference heath centre (vs heath centre)	0.49 (0.25)	0.64 (0.26)	0.54 (0.38)		2.53 (1.27)*		0.89 (0.36)		0.45 (0.28)		0.41 (0.16)**	0.50 (0.16)**
Age	0.97 (0.02)*	0.97 (0.01)*	0.94 (0.03)**	0.96 (0.02)**	0.89 (0.04)***	0.92 (0.02)***	1.03 (0.02)		0.96 (0.03)		1.01 (0.02)	
Male (vs female)	0.82 (0.27)		0.80 (0.32)		1.32 (0.68)		1.03 (0.32)		3.37 (1.62)**	2.36 (1.01)**	1.57 (0.47)	1.74 (0.44)**
Number of dependents	1.08 (0.05)*	1.07 (0.04)**	0.99 (0.05)		1.01 (0.08)		1.06 (0.03)*	1.08 (0.03)***	1.09 (0.04)**		1.05 (0.03)	1.04 (0.03)
Married (vs not married)	0.92 (0.11)		0.95 (0.17)		0.88 (0.20)		1.08 (0.13)		1.34 (0.24)		0.84 (0.10)	
University (vs school education)	0.83 (0.33)		0.84 (0.38)		1.32 (0.79)		0.93 (0.27)		1.02 (0.43)		1.28 (0.36)	
ASSP programme	1.55 (0.64)		0.41 (0.17)**	0.48 (0.17)**	2.47 (1.13)**		1.51 (0.44)		1.74 (1.05)		1.98 (0.64)**	1.80 (0.49)**
Receives any government pay^a^	0.20 (0.08)***		0.95 (0.39)		4.11 (2.37)**	2.76 (1.33)**	0.55 (0.17)*	0.51 (0.15)**	^b^		1.68 (0.47)*	1.92 (0.50)**
Receives user fees	–	–	0.89 (0.41)		–	–	1.32 (0.41)		–	–	–	–
Receives informal payments	–	–	–	–	–	–	1.32 (0.48)		–	–	–	–
Receives payment from private practice	–	–	–	–	–	–	3.00 (1.56)**	2.64 (1.21)**	–	–	–	–
Receives performance payments	–	–	–	–	–	–	0.67 (0.21)		–	–	–	–
Constant	14.87 (20.39)**	4.24 (2.58)**	2.54 (3.75)	0.77 (0.65)	0.27 (0.48)	0.12 (0.13)*	0.03 (0.03)***	0.18 (0.13)**	0.00 (0.00)***	0.00 (0.00)***	0.15 (0.14)**	0.19 (0.13)**
Pseudo *R* ^2^	0.15	0.07	0.15	0.10	0.24	0.18	0.16	0.15	0.46***	0.44***	0.13	0.11
Model *χ* ^2^	44.56***	21.42***	35.55**	29.59***	42.99***	29.19***	53.16***	44.09***	77.28	47.64	47.41***	52.87***
Number observations (*n*)	333	391	332	405	286	329	326	367	266	266	333	372

*p ≤ 0.1; **p ≤ 0.05; ***p ≤ 0.01

^a^Government pay = salary and/or occupational risk allowance

^b^The small number of observations meant receipt of government payments could not be included in the model for performance payments

During in-depth interviews, all nurses reported receiving income from user fees. The process for allocating user fees within the facility was usually overseen by the head of the facility. However, record keeping was often poor meaning the total revenue generated from user fees and allocation process was unclear to some nurses.

The way in which we divide (user fees)…I don’t know if I receive the same thing. The IT (head nurse) and IA (assistant nurse) and me, I don’t know if they give the same thing. They give it to me, I sign, that is all.

Female, 60 years

As shown in Table 5, nurses were more likely to receive informal payments if they were not based in Equateur. Staff at facilities with a higher number of personnel were less likely to report receiving informal payments (OR 0.67, *p* = 0.07) and facilities supported by ASSP reported a lower likelihood of informal payments (OR 0.48, *p* = 0.039). Older workers were less likely to receive informal payments (OR 0.96, *p* = 0.08).

The qualitative findings revealed that many nurses were reluctant to charge informal fees, as patients were usually so poor that they struggled to pay user fees. Nurses were less likely to charge informal fees where communities were well informed about the facility user fee tariff, for example, in ASSP areas where community health committees (CODESAs) and facilities were involved in setting and publicising tariffs, meaning nurses could be chastised by the public for asking for informal payments.

*…*everyone knows, that if you are going to ask for something someone will tell on you, you will be humiliated all the same, instead of asking, you must leave it.

Male, 42 years

Receiving income from private practice was more common in urban than rural areas (OR 2.44, *p* = 0.029) and facilities close to the village. Older workers were also less likely to receive income from private practice (OR 0.92, *p* < 0.000) and staff receiving government payments were more likely to receive income from private practice (OR 2.76, *p* = 0.036). Workers in Kasai Occidental and Maniema were more likely to work privately compared to those living in Equateur.

Reasons given by nurses for not engaging in private practice during interviews included being based far from private clinics, a perceived reduction in job security, and risks of losing the chance of becoming registered with the state and therefore receiving future government pay. However, some admitted that those currently receiving government pay may have been more likely to work privately to supplement their income, which is consistent with the quantitative analysis. Some nurses voiced that the private sector was superior to the public sector, as it was better resourced, staff were better paid and more motivated. However, many criticised the private sector for poor management and a lack of accountability, with patients not being treated according to best practice, and no focus on preventative care.

In the private (facilities) the staff are self-directed but they do not have any sanctions, they behave as they want. But with us here, the hierarchy demands explanations, there is monitoring

Female, 38 years

Staff at facilities with a higher number of personnel were less likely to report receiving per diems (OR 0.72, *p* = 0.019). Nurses in reference health centres were less likely to receive per diems (OR 0.45, *p =* 0.032) than those in health centres, while nurses in facilities far from the village were less likely to earn performance payments (OR 0.79, *p =* 0.001) than those near to the village. Facilities supported by ASSP reported a higher chance of receiving per diems (OR 1.80, *p =* 0.031) as well as workers receiving government payments (OR 1.92, *p* = 0.012). Males and workers in facilities offering a higher number of services were also more likely to receive performance payments and per diems.

During interviews, nurses indicated a preference for government payments over performance payments from development partners as they saw these as more stable and less transient sources of income.

Because the state, I could stay with the state until death. But the partner, will always be there for a term of 5 years

Male, 30 years

Some of the nurses interviewed felt that per diems were not allocated fairly.

Ah, it is not well managed (per diems), if someone tells us there is maternity training, it is one person who can go, from the other side it is the IT (head nurse) and IA (assistant nurse), so we others…nothing!

Female, 37 years

Workers reporting income from non-clinical activities were more likely to report income from private practice (OR 2.64, *p =* 0.035), be based in rural areas (OR 0.51, *p* = 0.025) and have a higher number of dependents (OR 1.08, *p* = 0.008). However, workers receiving government payments appeared to be less likely to receive income from non-clinical work (OR 0.51, *p* = 0.02).

Some nurses reported in interviews that those receiving government payments were actually more likely to undertake non-clinical activities, as they knew they would receive their government payments whether they worked in the facility or not but this was inconsistent with the quantitative findings. One worker admitted not coming to work to enable cultivation of crops to earn more income. Nurses who did not engage in supplementary non-clinical activities indicated this was due to a lack of time or an absence of the necessary resources or start-up capital.

They become negligent…you see, at the end of each month, you go to the bank, you see (them) but you go to the office and there is no-one working. They end up perhaps going to sell things, but at the end of the month, they will go to get their money.

Female, 30 years

### Total remuneration

In Province Orientale (*β* = −0.47, *p =* 0.032) and Maniema (*β* = −1.26, *p* < 0.000), nurses had lower levels of total income than nurses in Equateur. Males earned more income overall than females (*β =* 0.21, *p =* 0.05). Receipt of each income source was associated with a higher overall total income, with the exception of informal payments and payments from private clinical work (Table 6).

**Table 6:** OLS model for total remuneration

Explanatory variables	Coefficient (SE)	
	Full model	Reduced model
Years in position	−0.01 (0.01)	
Kasai Occidental (vs Equateur)	−0.37 (0.19)*	−0.27 (0.17)
Kasai Orientale (vs Equateur)	−0.11 (0.30)	−0.07 (0.28)
Maniema (vs Equateur)	−1.27 (0.26)***	−1.26 (0.18)*** (*p <* 0.001)
Province Orientale (vs Equateur)	−0.74 (0.24)***	−0.47 (0.22)** (*p =* 0.032)
Population served	0.00 (0.00)	
Total personnel	−0.01 (0.08)	
Urban (vs rural)	0.22 (0.26)	
Number of services	0.06 (0.04)	
Distance of facility from village	0.01 (0.02)	
Reference heath centre (vs heath centre)	−0.26 (0.21)	
Age	0.01 (0.01)	
Male (vs female)	0.26 (0.13)**	0.21 (0.12)*
Number of dependents	−0.01 (0.02)	
Married (vs not married)	−0.03 (0.04)	
University (vs school education)	0.11 (0.12)	
Supported by ASSP programme	−0.13 (0.18)	
Receives salary	0.73 (0.14)***	0.79 (0.12)*** (*p <* 0.001)
Receives occupational risk allowance	0.81 (0.15)***	0.70 (0.12)*** *p <* 0.001
Receives performance payment	0.59 (0.18)***	0.77 (0.15)*** *p <* 0.001
Receives user fees	0.65 (0.20)***	0.75 (0.17)*** *p <* 0.001
Receives informal payments	0.01 (0.17)	
Receives income from private clinical work	−0.01 (0.25)	
Receives supplemental income	1.03 (0.13)***	1.00 (0.10)*** *p <* 0.001
Receives per diems	0.20 (0.13)	0.20 (0.11)*
Constant	2.45 (0.42)***	2.91 (0.22)***
*R* ^2^	0.48***	0.44***
Number observations (*n*)	268	328

*p ≤ 0.1; **p ≤ 0.05; ***p ≤ 0.01

## Discussion

Nurses constituted the majority of personnel in both primary and secondary care and were the main focus of the study. The high variability in the amounts earned from each income source may be due in part to the fact that nurses make up a fairly heterogeneous group of different grades and levels of educational attainment.

Only a minority of nurses received a government salary, and a higher proportion received occupational allowances, with uncertainty regarding the timing and extent of payments. Part of the reason for the difference between government payments is that they are managed by two different Ministries; the Ministry of Public Sector Reform is responsible for the payroll while the occupational risk allowance is issued using the “declarative list” controlled by the Ministry of Health. Several bottlenecks have also been identified in the budget process which can result in a low execution rate of the allocated funds [15].

The extent to which either type of government payment was received varied across the provinces, likely due to differences in the available government budget for remuneration and a lack of transparency in the allocation of funds by provinces; the majority of the executed funds by province are usually untraceable (H Colquhoun, pers comm). A recent study of Katanga, South Kivu and Kasai Oriental provinces found the allocation and execution of the health budget was inequitable and not based on any pre-defined criteria (e.g. per capita and health indicators) [16]. The occupational allowance also constitutes a lower amount than the salary, potentially allowing more nurses to be paid within the allocated budget. A greater proportion of the allocated health budget goes towards the occupational risk allowance than salaries [11]. A repeat census of workers is also needed in order to identify nurses who have more recently started working in facilities and ensure they are paid. Nonetheless, the study found that receiving government payments sometimes had the unintended consequence of giving workers the freedom to work in private practice or non-clinical activities, potentially displacing them from their duties in public facilities.

User fees were commonly reported, representing a substantial share of total income consistent with Bertone et al. [9], but were also highly variable, depending on tariffs and case load. Informal payments appeared infrequent and small, particularly in the ASSP area which aims to improve health service accountability in relation to charges levied and payments received. Where paid, performance payments tended to be comparable in their amount to the occupational risk allowance and income from user fees, and vary by geographic area depending on donor and NGO presence. Health workers in Maniema were more likely to receive performance payments as they were still receiving payments from the ASSP programme at the time of the survey, although this was being phased out. Nurses in Maniema were also less likely to receive salaries. As the government budget is fungible, it is possible that the government prioritises the allocation of salaries on areas not supported by donor programmes. Health workers may also be less likely to push for inclusion on the payroll if they are receiving an income which substitutes their salary [17]. Nonetheless, workers tended to value government payments more than performance payments, similar to the findings of Fox et al. [6].

Per diems were received by just over half of nurses but contributed little to total income, consistent with Bertone et al. [9]. Per diems were sometimes perceived to be unfairly managed. We found evidence of gender discrimination in the allocation of per diems as well as performance payments, with male nurses being significantly more likely to receive these. Several studies in low-income countries have demonstrated how the mismanagement and abuse of per diems and performance payments can contribute to a negative organisational culture, on account of the tensions they create [18–20]. Care is needed to ensure such payments are distributed equitably across facility personnel and the same staff are not benefitting each time. Payments for overtime were not examined here but were found to be largely irrelevant by Bertone et al. in this context [9].

Less than 10% of workers conducted private clinical work, which corresponds with Bertone et al. [9] and this was more common in facilities close to villages and in urban areas similar to evidence from other countries [8, 10]. Nurses were more likely to engage in dual practice if they received income from the government. Nurses not receiving government payments thought it would be too risky to work in private facilities as it could jeopardise their chances of gaining registration.

Almost half of all workers engaged in non-clinical activities to supplement their income, higher than observed by Bertone et al. [9]. Agricultural practices were the most common which may be because the survey sampled predominantly in rural areas. These activities were sometimes carried out during working hours, which would impact on service delivery.

In terms of the total amount of income gained, differences were driven by both individual and provincial characteristics, again similar to Bertone et al. [9]. Males were more likely to receive a higher total income than females, indicating a gender inequity in receipt of income [21], while workers in Equateur were more likely to earn more than those in Province Orientale or Maniema. Unlike Bertone et al. [9], we did not find any association between facility characteristics and total income; however, their study included a wider variety of facilities.

This study attempts to shed some light on the complex puzzle of how to incentivise vital health workers in hard-to-reach areas in the context of a fragile state. Future policies should try to address some of the unacceptable inequalities related to gender or provincial location. There is low satisfaction with the amount received from formal sources, necessitating an increase in the current wage allowance, as well as perhaps the provision of non-financial incentives such as training and opportunities for career progression in order to effectively retain the workforce.

There were several limitations to this study. Firstly, the health worker survey was limited to those available on the day of the survey and does not capture the views of those absent. Secondly, workers may have under-reported or inaccurately recalled their income [22]. As robust documentation of health worker incomes does not exist in the DRC, it was not possible to validate estimates. Due to resource constraints, qualitative interviews could only be conducted in one of the five provinces and so we were unable to identify reasons for the provincial variation observed. The qualitative interviews preceded the analysis of the quantitative data, and so the quantitative findings could not be discussed during the interviews. A further study which uses the findings of the quantitative analysis as a basis for interviews may allow for more nuanced views. Finally, the facilities sampled represent 2.3%^4^ of the overall number of state primary care health centres and therefore the results are not necessarily representative of the provinces as a whole.

## Conclusions

In this study, we found that few workers received a government salary but a larger proportion received government payment through the occupational risk allowance. Often, there was a mixture of individual, facility and geographical factors associated with the receipt of various income sources. Greater co-ordination is therefore needed between all partners involved in the remuneration of workers in order to design more effective financial incentive packages, reduce the fragmentation of incomes and improve transparency in the payment of workers in the DRC.

## Appendix 1

**Table 7:** Hypothesised relationship of independent variables with income sources

Variables	Hypothesised relationship with income sources
Age	The older the worker, the more likely they are to gain income as elders are respected in DRC (Oppong & Woodruff, 2007). In addition, older workers will have been working for longer and may be paid more based on their experience.
Sex	Globally, while women comprise the majority of employees in the formal health system, they are usually less likely than men to hold senior roles, which tend to receive more pay (World Health Organization, 2010). In a study in Sierra Leone, for certain cadres, women received significantly less salary than males (Witter et al., 2015). In addition, according to the latest Gender Equality Index, DRC was ranked near the bottom (United Nations Development Programme, 2014). Therefore, it will be interesting to examine whether gender inequality also exists in the receipt of certain sources of income (e.g. user fees) when health worker position and education is controlled for. A study in Tajikistan has shown that women are equally as likely as men to charge informal payments once other factors have been controlled for but this has not been explored in other contexts (Dabalen & Wane, 2008). The same study also showed that women were less likely to work outside of the health facility than men.
Number of dependents	There is some evidence that in DRC, those that earn more have a higher number of dependents and so the number of dependents may increase as overall income increases (Weijs, Hilhorst, & Ferf, 2012).
Urban-rural status	Urban areas have a higher population density and so income from user fees may be higher. There are also large discrepancies in access to healthcare between urban and rural areas, with access being higher in urban areas, which may also affect income gained from user fees (World Bank, 2008). Opportunities to receive income from dual practice may be greater in urban areas compared to rural areas, as was observed in Zimbabwe (Chirwa et al., 2014). In addition, a study in Malawi revealed that urban health workers had higher monthly household incomes compared to their rural counterparts (Bowie, Mwase, & Chinkhumba, 2009).
Province	There are large differences in poverty between provinces in the DRC which may have implications for both formal and informal fees charged to patients (Moummi, 2010; United Nations Development Programme, 2009). Equateur is comparatively poorer than the other provinces that have been sampled. According to a recent study, there are wide provincial disparities in domestic public spending on health services, which may affect the amount of government payments received by workers (UNICEF, 2015; World Bank, 2008).
Total number of staff delivering healthcare present on the day	There is some evidence that facilities with more staff receive more income than understaffed facilities (Murro & Pavignani, 2012). On the other hand, income from user fees may be reduced as they are usually divided among workers at the end of the month. Having a high number of personnel may result in lower amounts being received by each staff member (Bertone & Lurton, 2015).
Number of services offered	Increasing the number of services available to a population is one way of improving access (Gulliford et al., 2002). This improved access may be reflected in increased utilisation rates resulting in higher incomes from user fees.
Distance of the facility from the village	Evidence has shown that distance travelled by patients is a key determinant of the utilisation of health services, and so may impact on the amount of user fees collected at facilities (Shannon, Bashshur, & Metzner, 1969).
Education	The level of education will vary by position and within positions. Doctors should hold a seven-year university degree, while the education of nurses depends on their grade; it varies between two years of secondary school to a three year university degree (Yngfors & Andersson, 2010). The difference in grade (and therefore education) is reflected in the payment of salaries.
Marital status	Several wage determination studies have found a positive wage effect of marriage even when other variables such as productivity and hours worked have been controlled for (Korenman & Neumark, 1991; Pfeffer & Ross, 1982; Kalachek & Raines, 1976; Hill, 1979).
Years in position	The longer a worker has been in their position, the more likely they are to receive a salary as they may have been identified in the last comprehensive health worker census in 2006. This census aimed to ensure workers were correctly registered on the government payroll.
Type of facility	Reference facilities are bigger, offer more services and serve a greater population compared to health centres. Therefore, income opportunities may be different within each.
Total population of village	User fees and therefore total income are influenced by demand factors such as the total population eligible to access healthcare.
Presence of ASSP programme	The programme implemented a subsidised user fee policy which would have influenced the amount of income gained from this source. In addition, the programme does not supply performance payments and was even phasing out performance payments in the province of Maniema provided by a previous health programme at the time of the survey. Finally, the programme has a mandate to strengthen the accountability of health services to the community; it would therefore be expected that informal payments would be less common in ASSP sites.

## Appendix 2

**Table 8:** Regression diagnostics

Model	Income sources	Regression diagnostics	Assumptions tested
Logit model	All sources of income	Ramsey RESET test	Functional misspecification
		Hosmer-Lemeshow test	Goodness of fit
Ordinary least squares*	Total income, salary, occupational risk allowance and user fees	Shapiro-Wilk test	Normality of residuals
		Ramsay RESET test	Functional misspecification
		Breusch-Pagan/Cook Weisberg test	Homoskedasticity
		VIF test	Multicollinearity

*For OLS, log of positive values was used

## Abbreviations

ASSP: Accès Aux Soins de Santé Primaires (Access to Primary Health care)
CODESA: Community health committees
DFID: Department for International Development
DRC: Democratic Republic of Congo
FC: Congolese Francs
NGO: Non-governmental organisation
OR: Odds ratio
PPS: Probability proportional to size
USD: United States dollars

## Footnotes

1 At the time of the study, DRC was composed of 11 provinces which have since been divided into 26 provinces.

2 Informal payments defined as payments made by patients outside of official channels

3 Exchange rate as of 26 June 2015 using FOREX currency converter.

4 Calculated using population data obtained from the Direction d’Etudes et Planification, Ministère de la Santé Publique in October 2013.
